# Evaluation of apically extruded debris following glide-path preparation with different file systems

**DOI:** 10.6026/973206300200683

**Published:** 2024-06-30

**Authors:** Gurudutt Nayak, Philip Pradeep, A. R. Vivekananda Pai, Neha Singh, Anik Banerjee, Chinmay Vartak, Rahul Sharma

**Affiliations:** 1 Department of Conservative Dentistry and Endodontics, Mansarovar Dental College, Bhopal, Madhya Pradesh, India; 2 Department of Conservative Dentistry and Endodontics, Faculty of Dentistry, Manipal University College Malaysia, Melaka, Malaysia; 3 Department of Conservative Dentistry and Endodontics, College of Dental Science & Hospital, Rau, Madhya Pradesh, India

**Keywords:** Debris extrusion, glide-path, manual instrumentation, nickel-titanium rotary file

## Abstract

The amount of apically extruded debris following glide-path preparation of mesial root of 120 freshly extracted human mandibular molar teeth using Senseus ProFinder files, PathFile, G-Files, Scout-RaCe files, HyFlex glidepath files and V glide-path two file system is of interest. The Eppendorf tubes were used as test equipment for collecting debris and the average weight of the debris was measured using an electronic micro-balancing system. It was observed that regardless of the file system utilized, debris was expelled from the apex. The G files resulted in a lower quantity of debris being extruded (0.070 ± 0.002 mg). In contrast, the V glide-path two file system exhibited the highest amount of debris extrusion (0.110 ± 0.004 mg) compared to all other file systems.

## Background:

The primary objective of root canal shaping and cleaning is to preserve the canal's original shape while minimizing errors [1]. Several tools help facilitate this procedure; however, a prevalent problem with nickel-titanium (NiTi) rotary instruments is their susceptibility to fracture caused by bending or shear stress. Curved canals can experience this phenomenon due to recurrent stressors [2, 3-4]. To prevent errors like ledge development, coronal enlargement, and creating glide paths early on, it is recommended that tool life be extended, and outcomes improved [5]. A glide route in endodontics refers to a uniform pathway that extends from the canal's opening to its endpoint [6]. The procedure involves clinical techniques to prepare the root canal before using larger shaping files [7]. Typically, it starts with a loose No. 10 file, progresses to a loose No. 15 file, and may end with a No. 20 file [6, 7, 8-9]. It is suggested that the canal should be one size larger than the initial rotary file [10]. Several file systems for glide-path prep are available. Senseus ProFinder (Dentsply Maillefer, Ballaigues, Switzerland) hand files come in stainless steel with 10, 13, and 17 tip diameters. Lengths include 18 mm, 21 mm, and 25 mm. Made through wire torsion, the file is characterized by a square cross-section with a pointed end that gradually narrows from 0.02 to 0.01, enhancing buckling resistance. The files' 65° non-cutting tip aids in navigating narrow calcified canals without compromising strength. Variable and minimal taper behind the tip allows flexibility and better tactile feedback, perfect for initial canal work [11]. Introduced in 2009, PathFile by Maillefer (Dentsply, Ballaigues, Switzerland) was the first NiTi rotary system for glide-path prep. It comprises three instruments: PathFiles: #1 is purple, # 2 is white, and # 3 is yellow with ISO-tip sizes for these PathFiles are 13, 16, and 19. With a square cross-section and 0.02% taper, these files are cyclic fatigue-resistant, flexible, and effective for cutting. Their non-cutting tip (50-degree angle) minimizes ledge formation. Available in 21, 25, and 31mm lengths, PathFiles are suggested for utilization post #10 hand file exploration, utilizing a motor configuration of 300 rpm and high torque of 5-6 N/cm2 [2]. The G-Files instruments (Micro-Mega in Besancon, France) were first released in 2011. They include two types of files, namely G1 and G2, with ISO tip sizes of 12 and 17, respectively. The 21mm, 25mm, and 29mm lengths files feature an asymmetrical non-cutting tip for efficient canal navigation. With a 3% taper, three radii blades for debris removal, an electro-polished surface, and a recommended 400 rpm setting with 1.2N/cm torque, these files are optimal for utilization once the canal has been explored to its working length with a size 10 hand file [2]. The ScoutRace files (FKG Dentaire in La Chaux-de-Fonds, Switzerland) are available in ISO tip sizes of 10 (purple), 15 (white), and 20 (yellow), all with a 2% taper. The triangle files undergo electropolishing to eliminate any imperfections caused by grinding. They are equipped with a noncutting tip, RaCe flute design, and alternate cutting edges. Following the initial exploration with a size 06 or 08 K file, the files should be operated at 800 rpm and 0.3 N/cm torque. The files are available in 21, 25, and 31mm lengths [2. The HyFlex glidepath file system (Coltene-Whaledent in Altstatten, Switzerland) includes two # 15 (white) files with ISO 15 tip size and 1% or 2% tapers and one #20 (yellow) file with an ISO tip 20 size and 2% taper. Available in 21, 25, and 31 mm lengths, these NiTi controlled memory (CM)-wires instruments are best used at 300 rpm and 2.4N/cm torque [12]. The V glide-path two-file system (SS white in Lakewood, New Jersey, USA) includes V03 and V04 rotary instruments. They have ISO tip sizes 13 and 17, with 3% and 4% tapers. Lengths available are 21mm and 25 mm. The manufacturer recommends using these files following the initial canal exploration with a # 10 K file. The recommended motor setting is 300rpm with a torque of 1.2N/cm. Files have a non-cutting tip with a variable taper to preserve dentin in the coronal third and a variable pitch design to reduce the screw-in effect [13]. An inherent disadvantage of root canal instrumentation is the expulsion of debris and microorganisms into periradicular tissue [14,15]. Vander Visse and Brilliant [16] were the pioneers in quantifying the extent of debris expelled in the apical direction. It was found that using irrigation during instrumentation led to extrusion, but instrumentation without irrigation did not result in significant debris accumulation. Research shows that rotational action techniques minimize apical debris compared to push-pull motion. Rotational methods, whether engine-powered or with balancing force methods are suggested to draw debris into instrument flutes, causing coronal exits [15]. The expelled remnants, referred to as the necrotic debris worm, can induce periapical inflammation and postoperative flare-ups characterized by symptoms such as discomfort and swelling [17]. Flare-ups, which happen in 1.4-16% of root canal treatments, are caused by root canal-related substances that stimulate intense inflammatory reactions extending beyond the apical foramen [15,18]. While there have been studies examining debris extrusion following canal preparation using various shaping file systems alongside or without glide-path preparation [19, 20, 21, 22, 23-24], a thorough search in PubMed revealed only one study [25] of debris extrusion following preparation of glide-path with different single-file systems. Therefore, it is of interest to assess and evaluate the apically extruded debris following glide-path preparation employing different multiple glide-path file systems.

## Methods and Materials:

## Sample size calculation:

With a confidence level of 95%, a confidence interval of +/- 5%, and a standard deviation of 0.5, the sample size for the study was determined to be twenty in each group. This was determined by utilizing the Cochran formula.

## Sample selection and specimen standardization:

One hundred and twenty freshly extracted mandibular molars removed for periodontal problems were collected for this investigation. The teeth were sterilized in a solution containing 0.5% chloramines T trihydrate for 7 days. The ultrasonic scaler was used to clear hard deposits and adhere tissue tags from the external root surfaces of experimental teeth. The teeth were later preserved in a physiological saline solution until utilized. The molars were radiographed from the buccal to the lingual side and from the mesial to the distal side. Only mesial roots exhibiting two distinct canals and two independent apical apertures were selected. The curvatures of these roots were quantified using the Schneider [26] technique and only those with curvatures ranging from 0° to 10° were chosen. To achieve a root canal length of 12±1 mm with accuracy, a diamond disc (BEGO GmbH & Co. KG in Bremen, Germany) was employed to separate each tooth's crown piece and distal root at the cementoenamel junction. The apical gauging procedure involved K-files of sizes 06, 08, and 10, (Dentsply Maillefer in Ballaigues, Switzerland). The study specifically examined teeth with a size 08 K-file that was hardly visible at the tip and a size 10 K-file that snugly fit at the working length. Furthermore, teeth that exhibited well-developed apices with unobstructed openings, absence of fractures, and absence of resorption both internally and externally, absence of decay on the roots, absence of blockages or calcification in the root canals, and absence of pulp stones were chosen. The study comprised 120 mesial molar roots that met the required criteria. The mesiobuccal canal was employed to assess the expulsion of debris. The working length was adjusted to be 1 mm less than the length at which a size 08 K file was observed at the apical foramen when viewed using a microscope (Labomed PRIMA DNT; Labo America Inc. in Fremont, CA) at a magnification of x25. All external surfaces of the tooth, except for a 1mm area surrounding the apical foramen, were covered with two layers of nail polish.

## Test apparatus:

A modified version of the test apparatus provided by Myers and Montgomery [27] was utilized to gather and assess extruded debris. The Eppendorf tubes (Eppendorf India Limited in Chennai, India) were used to set up the system for collecting the material extruding apically. Before gathering everything in the apparatus, the stoppers of the Eppendorf tubes were removed, and the initial weight of each tube was determined employing an analytical microbalance (AY 120 Analytic Balance, Shimadzu Corporation in Tokyo, Japan) with sealed housing and closed windows that offers an accuracy of +/- 0.0001 gm. Three readings were collected on average for each tube to minimize numerical inaccuracy. If three consecutive measures yielded significantly different values, the weighing process was maintained until three identical measurements were obtained, with the sole variation being the last digit deviating by 12. Then with a heated device a hole in the middle of the stopper on every Eppendorf tube was created to fasten the teeth. Afterward, the teeth were placed into the pre-cut holes in the stoppers and securely attached, utilizing cyanoacrylate adhesive up to the cervical level. Subsequently, the stopper was attached to every tube, and a 27-gauge bent needle was inserted close to balance the air pressure inside and outside the tube. The complete assembly was moved to a glass vial to prevent any interaction with the Eppendorf tubes during the process. Afterward, all vials were wrapped in foil made of aluminum to eliminate any possible prejudice by prohibiting the operator from visually detecting any debris during the procedure.

## Root canal instrumentation:

One hundred twenty samples were arbitrarily distributed into six groups (Table 1), each consisting of 20 samples (n = 20). The instrumentation system employed for group I consisted of stainless-steel hand files, whereas the other groups utilized rotary NiTiglide-path files.

The trajectory for Senseus ProFinder files was determined using the balanced force technique. All rotary glide-path files used in this experiment were operated using an X-Smart contra-angle handpiece with a 6:1 gear reduction electric motor with a torque limitation (Dentsply Maillefer in Ballaigues, Switzerland). The manufacturer's set specific torque limitation and rotation speed were utilized for every file system by accessing the programmable menu on the endo motor. A layer of ethylenediaminetetraacetic acid (EDTA) (Glyde File Prep; Maillefer, Dentsply, Tulsa in OK, USA) was applied to each file as a lubricant to facilitate smooth movement during mechanical instrumentation. Beforehand using rotary instruments, a size 10 K-file was initially employed to scout the canal to its working length. During the rotary instrumentation process, light pressure with glide-path files and back-and-forth motions with amplitude between 2 and 3 mm were employed. Twenty canals were preflared in each of the six groups that were evaluated. One file was utilized to prepare four canals. One operator conducted all the root canal preparation, while a second investigator, uninformed of the experimental groups, evaluated the extruded debris.

## Irrigation protocol:

The irrigation protocol was standardized for all specimens, using water that had been bi-distilled in a 2 ml disposable plastic syringe (DispoVan, Hindustan Syringes & Medical Devices Ltd. in Faridabad, India) attached with a 30-gauge close-end tip and a needle with a double side-port aperture (RC Twents, Prime Dental Products Pvt. Ltd. in Maharashtra, India). If any resistance was encountered during the process of instrumenting, the files were removed, and irrigation was performed before reusing them. After each instrument change, the root canals were rinsed with 2 ml of double-distilled water for 1 minute.

## Assessment of extrusion:

After the completion of the instrumentation, the stopper was removed with the tooth and needle. The root was flushed into the tube of Eppendorf using 1 mL of water that had been double distilled to collect the particles adhering to the root's surface. Afterward, the Eppendorf tubes were placed in a 70°C incubator for 5 days to aid in moisture evaporation before calculating the weight of the dried residue. Each collecting assembly underwent three consecutive weight measurements, and the resulting average value was recorded. The extent of debris that was extruded apically was calculated by reducing the mean weight of the Eppendorf tube carrying the dry dust from the standard weight of the pre-weighed Eppendorf tube utilizing the same analytical microbalance as before.

## Statistical analysis:

The data acquired underwent a statistical evaluation utilizing the SPSS version 26.0 software for Windows operating systems, (IBM SPSS Inc. in Chicago, IL, USA). Statistically significant was defined as a condition where the p-value is lower than 0.05, and the threshold for determining significance is set at 5%. The test known as Kolmogorov-Smirnov was used to verify the normal distribution of the data. A one-way analysis of variance (ANOVA) was employed to analyze quantitative variables, followed by a post hoc Tukey analysis.

## Results:

**Figure 1:**
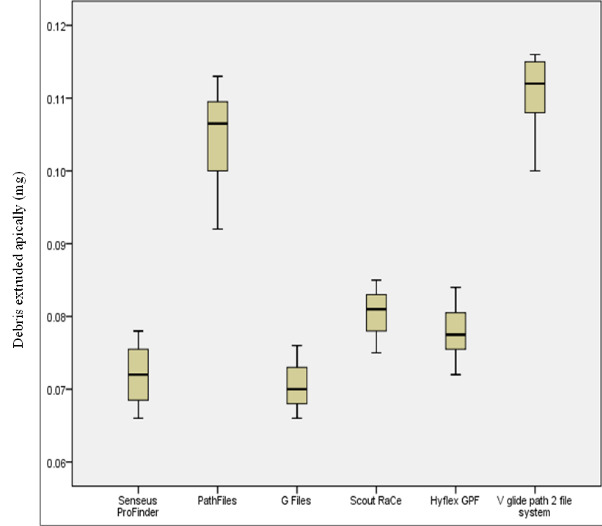
Box plots graphs illustrating the median, mean, minimum, and maximum values of the amount of apically extruded debris (mg) from each group tested.

Table 2 displays the precise weight of the extruded debris and multiple comparisons across different groups. The results of this investigation demonstrated that the expulsion of debris from the apex occurred irrespective of the type of equipment employed. Figure 1 shows a box plot graph for each group, showing the median and interquartile values. The G files generated less debris than all other file systems, exhibiting a mean value of 0.070±0.002 mg. Compared to the other examined systems, the V glide-path 2 file system showed the most outstanding amount of debris extrusion, measuring 0.110 ± 0.004 mg. Post hoc analysis revealed that there was no significant difference (p>0.05) in the amount of debris extruded apically between Scout-RaCe files (0.080 ± 0.002 mg) and Hyflex glidepath files (0.078 ± 0.003 mg), as well as between Senseus ProFinder files (0.072 ± 0.003 mg) and G-files (0.070 ± 0.002 mg). The current study identified statistically significant variations (p<0.05) between all the other file systems.

## Discussion:

The primary objective of the preparation of root canals is to preserve the initial canal structure while effectively cleaning and shaping the root canal system [1]. Most often, these goals have been accomplished due to the properties of NiTi rotary instruments. Nevertheless, these devices are vulnerable to torsional fractures due to intimate contact with the canal walls and bindings in the early stages of root canal preparation [4, 5]. In modern endodontics, a glide-path is strongly advised to lower the risk of torsional fractures. NiTi rotary instruments can be utilized safely by avoiding torsional fracture of the instruments and shaping aberrations through glide-path establishment and coronal enlargement [5]. The leading cause of postoperative pain and edema is primarily related to the host's immune response against extruded debris containing pathogens, excessive instrumentation, or obturating material that results from treatment procedures. During canal instrumentation and irrigation, the contents inside the canal, such as small pieces of dentin, fragments of dead pulp tissue, and bacteria, are pushed out as debris. This debris can potentially trigger inflammation in the area around the tip of the tooth root [29, 30]. Establishing a glide-path preparation before root canal preparation frequently results in decreased debris extrusion and postoperative discomfort [19].

An underlying drawback of shaping and cleaning root canals is the extrusion of debris and irritants from the apex. The quantity of debris and irrigant that extrudes periapically depends on many variables, including instrument size, instrument type, instrumentation technique, preparation endpoint, apical stop, glide-path, coronal enlargement, irrigation solution, and irrigation delivery system [14,16,21, 31, 32, 33- 34]. The present study compared the quantity of dentinal debris that was pushed out towards the apex after using various multi-sequence systems, such as Senseus ProFinder stainless steel hand files and rotary NiTi PathFile, G-Files, Scout RaCe files, HyFlex glidepath file, and V glide-path 2 file systems, for glide-path preparation. The study examined debris removal in curved root canals by selecting mesiobuccal canals with curvatures ranging from 0° to 10°. This was done to prevent complications such as loss of working length or inconsistent preparation and irrigation in curved root canals. To ensure constant removal of debris, differences in working lengths were eliminated, and a uniform shaping depth and irrigation penetration were maintained by standardizing the root canal length to imitate clinical settings. The study chose teeth based on apical gauging data to compare all file systems fairly. Double distilled water was utilized to prevent the expulsion of waste from particles in alternative irrigation agents. A mono jet irrigating syringe with a 30-gauge close-end tip and a double side-port opening needle were used to reduce the drive of the irrigant out of the canal, as opposed to conventional open-ended needles [34]. The measurements were conducted in a controlled, closed environment to prevent inaccuracies caused by the analytical balance's sensitivity to vibrations and humidity. In this research, all instruments used in glide-path preparation resulted in periapical extrusion of debris. G files showed the lowest extrusion of debris, followed by Senseus Profinder, HyFlexGPF, ScoutRace, and PathFile; on the other hand, the V glide-path 2 file system showed the highest mean apical extrusion of debris. The V glide-path 2 file system consists of V03 (pink) and V04 (white) rotary instruments with ISO tip sizes 13 and 17 and tapers of 3% and 4%, respectively. The V glide-path file has a greater taper of 0.04% at the apical 3 mm, which might be attributable to increased debris generation apically and extrusion periapically in the present investigation [35]. In the present study, a post hoc analysis demonstrated a statistically significant difference (p<0.05) in the quantity of debris extruded apically between the Path File group and the other treatment groups. PathFileare multi-sequence glide-path file system with a square cross-sectional area and a continuous taper of 0.02%. The file's cutting capacity is enhanced by its four cutting edges. The improved cutting capability of rotary NiTi instruments is frequently associated with an enhanced ability to remove debris, but it can also lead to an increased expulsion of debris. Additionally, a square cross-section will lead to a reduced chip space, which restricts their capacity to permit coronal clearance of debris, causing a piston-like movement that may cause debris extrusion [36].Multiple comparisons of the mean revealed significant differences (p=0.001) between Scout RaCe and other treatment groups except for the HyFlex GPF group (p=0.542). The ScoutRace alternating cutting edge with mixed pitch length may have also had a favorable impact on removing debris from the long pitch length [25]. The mean debris extrusion reported in the present investigation for the HyFlex GPF file system was low. During the instrumentation of all specimens, the spirals of HyFlex files unwind. This tendency can cause the instrument's cutting and cleaning effectiveness to decline. Consequently, fewer dentinal chips and debris were produced, and there was less extrusion of debris from the samples [37].

Comparing traditional hand instrumentation versus rotary instrumentation, it has been shown in earlier research that the former extrudes more debris [14, 38]. However, the set of hand files group in the on-going experiment had a lower average value of debris extrusion at the apex compared to the other rotating glide-path groups, except for the G File group. This can be attributed to the file design of Senseus ProFinder hand files used in the current study instead of K files used in previous studies [20, 22, 25, 38, 41]. These files come in sizes 10, 13, and 17, and they have a taper that approximately decreases from 0.02% at the tip of the blade to the shank, which is about 0.01% [11]. Tinaz *et al.* [39] observed that the quantity of debris extruded apically increased in teeth with larger apical patency. The largest Senseus ProFinder file has a smaller (0.17mm) tip size than any other glide-path files utilized in this investigation. Also, the balanced force technique was used in the present study, unlike the filing (push-pull) motion instrumentation technique used in previous studies, which pushed more debris beyond the apex [22, 28-38]. In the current experiment, the G glide-path system demonstrated the most petite average apical extrusion of debris. The unique geometric pattern of the G Files is the cause of this. The longitudinal view of the G Files displays cutting edges with three distinct radii relative to the canal axis, resulting in a pattern such as that of a snake. The angular displacement of the cutting edges leads to a diverse pitch across the entire length of the blade. Moreover, the files exhibit a diverse cross-sectional profile throughout its entire span. This contributes to a substantial and effective area for the upward movement of debris and, therefore, less debris extrusion [40].

Since no previous studies are available for comparison, this is the first study to assess multiple glide-path file systems for debris extrusion. Dagna *et al.* [41], in their study evaluating the apical extrusion of intracanal bacteria using different glide-path establishing methods, revealed that PathFile resulted in a more considerable number of bacteria being extruded compared to G Files. In contrast to the current study, Gunes and Yesildal Yeter [20] found no statistically significant distinction in the quantity of debris pushed out towards the apex between G Files and PathFiles when using the WaveOne Gold single-file reciprocating system to prepare curved root canals. This could be attributed to utilizing only two PathFiles, # 1 and 2, as the study was designed to permit glide-path preparation files with comparable tip diameters. Additionally, the number of files employed can impact the ejection of debris.

This study is limited by the absence of supplementary approaches that could replicate the in vivo model, such as using floral foams to simulate periapical tissues. These foams would act as a barrier to prevent the unintended release of debris and irrigants [34]. Instrumenting vital and non-vital teeth poses another limitation in assessing debris extrusion, with pulp stumps in vital teeth preventing debris from being forced out, unlike in necrotic teeth [42]. Dentin mineralization is a crucial factor, as it is lower in young teeth than adult teeth, making young teeth more susceptible to wear and extrusion. Curvature and many canals are other factors that can impact the final amount of apical extrusion [15]. A positive or negative pressure at the apex associated with normal or diseased periapical tissues is one of the other drawbacks of a clinical context determining the degree to which debris and irrigants extrude periapically [43]. Apical dentinal plug formation prevents over-instrumentation and debris extrusion, countering those shortcomings [44]. Despite the inherent drawbacks of the methodology suggested by Myers and Montgomery [27], it was selected for calculating debris due to its practicality and ability to facilitate comparisons of the quantities of debris expelled by each file.

## Conclusion:

Based on the limitations of this investigation, it was noted that all the file systems led to the expulsion of debris. The G Files produced less debris than the other file systems. The V glide-path 2 file system exhibited the greatest extrusion of debris. While the quantity of debris may be minimal, the debris that is initially expelled may have a higher toxicity level than debris expelled subsequently by the shaping instrument. Therefore, the expulsion of debris while preparing the glide-path would also have clinical significance. Subsequent research may provide a detailed analysis of the clinical impacts of glide-path preparation techniques and the possible consequences of debris extrusion.

## Figures and Tables

**Table 1:** Root canal instruments and manufacture details

**Serial Number**	**Name of Files**	**Manufacturer Details**	**Number (n)**
Group I	Senseus ProFinder files	Dentsply Maillefer in Ballaigues, Switzerland	20
Group II	PathFile	Dentsply Maillefer in Ballaigues, Switzerland	20
Group III	G-Files	Micro-Mega in Besancon, France	20
Group IV	Scout-RaCe files	FKG Dentaire in La Chaux-de-Fonds, Switzerland	20
Group V	Hyflex glidepath files	Coltene-Whaledent in Altstatten, Switzerland	20
Group VI	V glide-path 2 file system	SS White, Lakewood in New Jersey, USA	20

**Table 2:** Descriptive statistics regarding the amount of apically extruded debris (mg) from each group tested.

**Group**	**Mean ± SD**	**Minimum**	**Maximum**
Group 1: Senseus ProFinder	0.072±0.003a	0.066	0.078
Group 2: Path File	0.105±0.005b	0.092	0.113
Group 3: G files	0.070±0.002a	0.066	0.076
Group 4: Scout RaCe	0.080±0.002c	0.075	0.085
Group 5: Hyflex GPF	0.078±0.003c	0.072	0.084
Group 6: V Glide-path 2 system	0.110±0.004d	0.1	0.116
Values with different superscripts between groups indicate statistically significant differences (p < 0.05). SD = standard deviation.
